# Profiling Micro-RNA expression in patients with Primary Sjogrens Syndrome – contribution to disease pathogenesis

**DOI:** 10.1186/1753-6561-9-S7-A9

**Published:** 2015-10-27

**Authors:** Rena AL-Zubaidy, Joan Ní Gabhann, Qistina Pilson, Conor C Murphy

**Affiliations:** 1 Molecular and Cellular Therapeutics and RSCI Research Institute, Royal College of Surgeons in Ireland, Dublin 2, Ireland; 2 Department of Ophthalmology, Royal College of Surgeons in Ireland, Dublin 2, Ireland; 3 Department of Ophthalmology, Royal Victoria Eye and Ear Hospital, Dublin 2, Ireland

## Background

Sjogrens syndrome (SS) is a chronic autoimmune disorder, characterised by lymphocytic infiltration resulting in exocrine gland destruction and other extra-glandular manifestations [1]. Currently there is no definitive diagnostic test, and the immuno-pathology is not fully understood. Recently focus has shifted to investigating microRNAs (miRs) in an effort to understand the mechanisms contributing to disease pathogenesis. MiRs are short non-coding RNA sequences, which regulate gene expression post- transcriptionally [2]. Of note, miRs have been identified as key regulators of immune function in a variety of autoimmune conditions [2]. The hypothesis of this project is that alterations in expression of specific miRs which regulate genes relating to inflammation and immunity may play a role in the pathology of the disease.

## Methods

A microRNA screen had been conducted in peripheral blood mononuclear cells (PBMCs) from healthy controls and SS patients. From this screen a set of microRNAs were analysed by bioinformatics using online platforms including miRWalk, MiRDB and miRanda Tools. Primers were designed and optimised by PCR for the miRs and their predicted gene targets. As part of ongoing work in the lab PBMCs were isolated and transfected with a sequence that mimics miR-155 (a pro-inflammatory miR). The effect miR-155 on target genes was analysed qPCR.

## Results

Bio-informatic studies identified several novel miRs whose expression is altered in SS patients compared to healthy controls. Two miRs that were focused on were down-regulated in the patient sample and are hsa-miR-132-3p and hsa-miR-4535 and their target genes are ESRRG (Estrogen related receptor gamma) and ATRN (Attractin) respectively; and one up-regulated miR which is Hsa-miR-185-3p and its target gene is IRF5 (Interferon regulatory factor 5). Primers for these novel miRs and gene targets were optimised.

## Conclusions

Since these specific miRs have altered expression in SS patients, and have a direct effect on inflammatory genes, this indicates that these miRs may have biomarker potential and further study of these miRs might increase our understanding of the underlying pathology of Sjogrens syndrome and other autoimmune diseases.

## References

[B1] Bayetto K Logan RM Sjogren's syndrome: a review of aetiology, pathogenesis, diagnosis and management Australian Dental Journal 2010 55 1 47 20553243 10.1111/j.1834-7819.2010.01197.x

[B2] Sonkoly E Stahle M Pivarcsi A MicroRNAs and immunity: Novel players in the regulation of normal immune function and inflammation ELSEVIER 2008 18 2 131 140 18291670 10.1016/j.semcancer.2008.01.005

